# The Double-Edged Sword of Creative Control in Designer-AI Co-Creation with Design Experience as a Boundary Condition

**DOI:** 10.3390/bs16040570

**Published:** 2026-04-09

**Authors:** Wenyue Gong, Xiang Chen

**Affiliations:** School of Design, Jiangnan University, No. 1800 Lihu Avenue, Binhu District, Wuxi 214000, China; wyyjs2024@outlook.com

**Keywords:** creative control, psychological ownership, cognitive load, design experience, designer-AI co-creation, double-edged sword effect

## Abstract

As generative artificial intelligence (AI) becomes increasingly involved in creative processes, designers encounter a fundamental tension regarding creative control—the degree to which they dominate design direction and iterative decision-making when collaborating with AI. Existing theories offer contradictory predictions: self-determination and psychological ownership theories emphasize the benefits of control, whereas cognitive load theory highlights its cognitive costs. This tension remains empirically unresolved, particularly regarding how designer characteristics shape these competing effects. This study examines the dual-pathway mechanism linking creative control to design creativity and investigates the moderating role of design experience. A scenario-based between-subjects experiment was conducted with 226 designers and design students. Creative control exerted a positive indirect effect on design creativity through psychological ownership (effect = 0.16, 95% CI [0.09, 0.24]) and a negative indirect effect through cognitive load (effect = −0.07, 95% CI [−0.14, −0.02]), confirming the double-edged sword effect. Design experience strengthened the positive pathway while buffering the negative pathway. Creative control thus functions as a double-edged sword in designer-AI co-creation, with its net effect contingent on designer expertise. The results extend Conservation of Resources theory to human-AI collaboration contexts and inform the design of experience-adaptive AI-assisted systems.

## 1. Introduction

Designers across industries—from product design to architecture—are now routinely expected to integrate generative AI tools into their creative workflows. Yet this collaboration introduces a critical dilemma: how much creative control designers retain over AI-mediated processes directly affects the quality, originality, and personal meaning of their design outputs (Caramiaux et al., 2025). When this balance is poorly calibrated, designers risk either creative disengagement from excessive AI autonomy or cognitive overload from excessive manual oversight, both of which undermine innovation performance (Wadinambiarachchi et al., 2024). Without empirically grounded guidance, the design of AI-assisted tools will continue to rely on ad hoc assumptions about human-AI control allocation, potentially compromising the creative capacity of an entire professional workforce.

Generative AI is rapidly reshaping the creative process within the design industry. Tools like Midjourney, DALL-E, and Stable Diffusion—with their sophisticated visual understanding and generation capabilities—can now produce high-quality design alternatives within seconds. What once took designers hours of concept exploration now happens almost instantaneously (Verganti et al., 2020). Yet this shift represents more than accelerated workflows. It marks a qualitative change in design work itself, as designers’ core competencies increasingly shift from hands-on execution toward creative direction and aesthetic judgment (Caramiaux et al., 2025). The trajectory of AI in design has moved from basic drafting assistance through intelligent parameter optimization to what we might now call creative symbiosis (Yin et al., 2024). Whereas early computer-aided design (CAD) systems handled precise drafting with designers as the sole creative source, today’s generative AI actively proposes solutions and participates in creative decisions. This shift from “tool” to “collaborator” marks a genuinely new chapter in human-AI collaboration (Verganti et al., 2020).

Against this backdrop, design space exploration—the systematic process of generating, evaluating, and refining multiple alternatives (Koch et al., 2019)—is being fundamentally reshaped. This process sits at the heart of design innovation, directly influencing the quality and diversity of final solutions. Traditional workflows constrained exploration to what designers could cognitively manage within available time (Cross, 2023). Generative AI fundamentally alters this calculus: its capacity for algorithm-driven batch generation dramatically expands the scope of exploration. Yet this efficiency boost comes with a profound restructuring of collaborative relationships. As AI takes on more creative generation, designers increasingly become “guides and evaluators” rather than “direct producers” of creativity (Koch et al., 2019). This transition surfaces a critical question: how much creative control should designers maintain? Different allocations of control are likely to produce different effects on creativity.

Existing research has explored how generative AI affects design, yet most studies focus on tool functionality and interface design in relation to efficiency (Caramiaux et al., 2025; Koch et al., 2019). This approach has merit but also clear limitations—it often treats AI as a “black box” efficiency tool without adequately examining how control allocation affects designer psychology. On this point, the literature presents contradictory perspectives. On one hand, prior research suggests that maintaining autonomy and control over creative processes can foster intrinsic motivation and a sense of personal investment in outcomes (Hamrick et al., 2024; Gray et al., 2020). On the other hand, recent empirical work indicates that excessive control demands during AI collaboration may lead to design fixation and cognitive overload, constraining divergent thinking (Wadinambiarachchi et al., 2024; Yin et al., 2024). This theoretical tension implies that more control does not automatically produce better outcomes—high control may simultaneously generate psychological benefits and impose cognitive costs. This “double-edged sword” effect is precisely what this study investigates.

Given these gaps, this study aims to build and test a dual-pathway mediation model revealing how creative control influences design creativity in designer-AI co-creation. This study identifies creative control as an independent variable and examines how it activates two competing pathways: a “gain pathway” promoting creativity through psychological ownership, and a “loss pathway” inhibiting creativity via cognitive load. It also introduces design experience as a moderating variable. Research on expert-novice differences indicates that experienced designers possess more sophisticated knowledge structures and automated skills (Chen et al., 2022); however, this expertise has yet to be examined within the context of human-AI collaboration. These ideas are examined through a scenario-based between-subjects experiment (Aguinis & Bradley, 2014). This study contributes to the literature by offering a competing mediation perspective on creative control’s dual effects, with implications for differentiated control configurations in AI-assisted design systems. Specifically, this study addresses the following research questions: (1) Does creative control exert simultaneous positive and negative indirect effects on design creativity through psychological ownership and cognitive load, respectively? (2) Does design experience moderate these dual pathways?

## 2. Literature Review and Hypothesis Development

### 2.1. Psychological Ownership and Design Creativity

Creative control is defined as designers’ subjective sense of mastery over design direction, solution generation, and iterative decision-making within AI-mediated work. This concept draws on autonomy needs from self-determination theory (Ryan & Deci, 2000) while incorporating the specific characteristics of human-AI collaborative contexts—emphasizing designers’ felt dominance over creative decision-making. Conservation of Resources theory suggests individuals naturally seek to acquire and protect resources, which can be gained through some pathways while depleted through others (Hobfoll, 1989). This study hypothesizes that high creative control may simultaneously trigger two competing pathways: resource acquisition (psychological ownership) and resource depletion (cognitive load).

Psychological ownership describes a psychological state where individuals develop a sense of “this is mine” toward some target (Pierce et al., 2001). The construct emphasizes subjective possession from the individual’s perspective, distinct from legal ownership. Research identifies three core routes to psychological ownership: exercising control over the target, coming to know it intimately, and investing the self into it. Control is arguably the most direct pathway—when people can shape an object’s direction or development, they more readily perceive it as a self-extension (Pierce et al., 2003). This framework has been extensively validated in organizational behavior, where psychological ownership consistently promotes work engagement and innovation (Zhang et al., 2021). In creativity research specifically, Gray et al. (2020) found collective psychological ownership predicts early creative success, with ownership perceptions stimulating responsibility and motivational commitment toward creative outputs. Baer and Brown (2012) observed that when people view creative ideas as “their own children,” they show stronger protective intentions and improvement motivation. More recently, Hamrick et al. (2024) confirmed ownership’s facilitative effect on individual performance. In designer-AI collaboration, when designers lead the design direction, they should more readily perceive AI-generated solutions as extensions of their own creativity, translating ownership perception into enhanced creative motivation.

Based on the above reasoning, it is hypothesized that psychological ownership serves as a positive mediating mechanism linking creative control to design creativity (see Hypothesis 1 in Section 2.4).

### 2.2. Cognitive Load and Design Creativity

Sweller (1988) systematically developed cognitive load theory to explain working memory resource allocation and consumption. When overall load exceeds working memory capacity, information processing efficiency drops significantly (F. Paas et al., 2016). Creativity, as a higher-order cognitive activity, makes substantial demands on working memory. Divergent thinking requires extensive searches and associations within the problem space—an inherently resource-intensive process (Forthmann et al., 2019). Rodet’s (2022) experiments confirmed an inverted U-shaped relationship: moderate load activates cognitive systems while excessive load inhibits them. In AI collaboration contexts, high creative control means designers must dominate more decision stages—setting direction, evaluating AI outputs, making iterative choices. Each decision drains cognitive resources (Yin et al., 2024). Wadinambiarachchi et al. (2024) documented “design fixation” during AI collaboration—excessive focus on specific solutions that reduces capacity for exploring alternatives. This indirectly supports cognitive load’s inhibitory effect.

Therefore, it is hypothesized that cognitive load functions as a negative mediating mechanism in this relationship (see Hypothesis 2 in Section 2.4).

### 2.3. The Moderating Role of Design Experience

The cognitive differences between experts and novices are a classic topic in cognitive psychology. Chase and Simon (1973) demonstrated that experts possess richer domain-specific knowledge schemas, enabling them to process complex information through chunking and thereby reduce load. Chen et al. (2022) showed expert designers have more sophisticated knowledge structures for encoding and reorganizing information; novices must invest greater resources to handle identical tasks. The expertise reversal effect reveals an important nuance: instructional support effective for novices may backfire for experts who have already internalized knowledge into automated schemas (Kalyuga, 2007). Regarding the ownership pathway, experienced designers should effectively convert control into ownership benefits. Ozkan and Dogan (2013) found experts rapidly identify connections between solutions and their creative intentions, while novices need more cognitive effort for such analysis. When experienced designers lead design direction, they can more deeply recognize their contributions, generating stronger ownership perceptions. Regarding cognitive load, experienced designers can buffer associated costs. Lawson (2005) noted that design expertise fundamentally involves developing automated “procedural knowledge,” allowing many decisions to proceed without deliberate thought. When experienced designers maintain high control, automated schemas make decision-making relatively effortless; novices, lacking such structures, remain more vulnerable to overload.

Drawing on this reasoning, it is hypothesized that design experience differentially moderates the two pathways—strengthening the ownership pathway while buffering the cognitive load pathway (see Hypotheses 3 and 4 in Section 2.4).

### 2.4. Theoretical Model Integration

**Figure 1:**
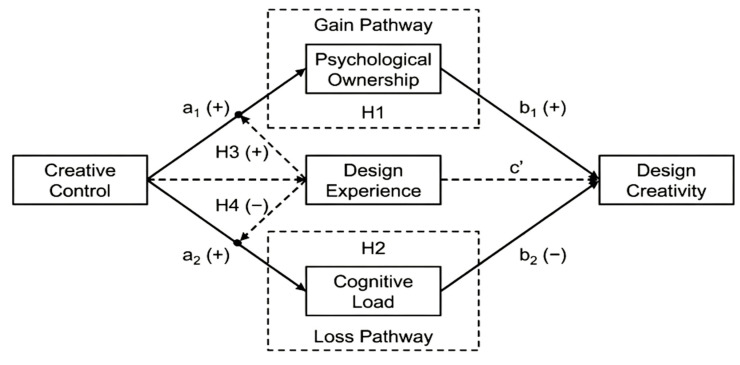
Theoretical Model.

Integrating the theoretical logic outlined above, the present study constructs a moderated dual-pathway mediation model, as illustrated in Figure 1. Conservation of Resources theory provides an integrative framework for understanding the dual effects of creative control (Hobfoll, 1989): high creative control simultaneously activates two pathways—resource acquisition (psychological ownership) and resource depletion (cognitive load)—which exert competing influences on design creativity. Design experience, serving as a boundary condition, further reveals the heterogeneity of the dual-pathway effects.

Accordingly, the following four hypotheses are proposed:

**H1:**  *Psychological ownership positively mediates the relationship between creative control and design creativity.*

**H2:**  *Cognitive load negatively mediates the relationship between creative control and design creativity.*

**H3:**  *Design experience positively moderates the relationship between creative control and psychological ownership, such that the positive effect of creative control on psychological ownership is stronger for highly experienced designers than for their less experienced counterparts.*

**H4:**  *Design experience negatively moderates the relationship between creative control and cognitive load, such that the positive effect of creative control on cognitive load is weaker for highly experienced designers than for their less experienced counterparts.*

## 3. Research Methods

This study adopts a quantitative, explanatory research approach grounded in the positivist paradigm. In terms of scope, it is an inferential study aimed at testing causal hypotheses regarding the psychological mechanisms underlying designer-AI co-creation. The research follows a hypothetico-deductive approach, deriving predictions from established theories and testing them empirically. In terms of its purpose, this is an applied study with implications for the development of AI-assisted design systems. It employs a cross-sectional, single-factor between-subjects experimental design, with primary data collected through an online scenario-based questionnaire administered via the Wenjuanxing platform. Data analysis combines confirmatory factor analysis for measurement validation with bootstrapped mediation and moderation regression analyses (PROCESS macro) for hypothesis testing. The following subsections detail the research design, sampling, experimental materials, variable measurement, and data analysis strategy.

### 3.1. Research Design

The present study employed a single-factor between-subjects experimental design, manipulating creative control to create two conditions: high and low creative control. The rationale for selecting a scenario-based experiment over traditional survey methods lies in the following consideration: as a subjective experience, creative control measured solely through retrospective self-reports may face insufficient measurement validity and endogeneity risks of reverse causality—that is, designers who inherently hold positive attitudes toward AI collaboration may tend to report higher levels of control and creativity. Scenario-based experiments, by directly manipulating independent variable levels, enable clear causal inference while controlling for confounding factors (Aguinis & Bradley, 2014). The research context focused on designers using AI tools to explore the design space for smart speaker aesthetics. Design space exploration refers to the process by which designers systematically generate, evaluate, and iterate multiple design alternatives using various tools (Woodbury & Burrow, 2006), and is particularly critical in AI-assisted design. This context effectively simulates authentic human-AI collaborative design scenarios while facilitating the manipulation of high versus low levels of creative control.

### 3.2. Sample and Data Collection

The target population was defined as design practitioners and design students with experience in using design software and either familiarity with or prior use of AI-assisted design tools. Participants were recruited via the Wenjuanxing online survey platform between September and October 2024. The student sample was drawn from design-related programs at universities in Eastern China, including Jiangnan University and Nanjing University of the Arts (approximately 120 participants). The professional designer sample was recruited through design industry communities (e.g., Zcool) and design firms in the Yangtze River Delta region (approximately 120 participants). Participation was voluntary and uncompensated. The requirement for prior experience was intended to ensure that participants possessed basic familiarity with AI-assisted design interaction modalities, thereby enabling immersion in the experimental scenario. Sample size was determined through a priori power analysis using G*Power 3.1, based on a medium effect size (f^2^ = 0.15), significance level (α = 0.05), and statistical power (1 − β = 0.80), yielding a minimum required sample size of 200 participants. Considering the two-group experimental design and potential invalid responses, the target sample size was set at 120 participants per group, totaling 240 participants. This study protocol was approved by the Human and Social Sciences Research Ethics Committee of Jiangnan University (Approval No.: JNU-2024-IRB-038). All participants read the study instructions and signed electronic informed consent forms prior to the experiment, acknowledging their understanding of the research purpose, the principle of voluntary participation, and their right to withdraw. Data were anonymized before analysis, and raw data were used solely for the purposes of the present study. Upon completing the informed consent process, participants sequentially completed demographic information and design experience questionnaires, followed by random assignment to either the high or low creative control condition via the survey platform’s randomization function. After reading the corresponding scenario materials, participants completed measures of manipulation check, psychological ownership, cognitive load, and design creativity in sequence. The entire procedure required approximately 10–15 min.

### 3.3. Experimental Materials Design

The design of experimental materials is a core component of the present study. Participants in both groups read a scenario description depicting a designer using AI tools for design space exploration of smart speaker aesthetics. The high creative control condition emphasized designer dominance over design direction, with AI serving an assistive execution role; the low creative control condition emphasized AI dominance over the exploration direction, with the designer responsible for selection and refinement. To eliminate potential confounding effects arising from differences in information quantity, the two scenario descriptions were strictly matched in terms of word count, design task, and product type, with the sole difference being the allocation of control. The core differences and controlled elements between the two experimental conditions are presented in Table 1. The full text of both scenario descriptions is provided in Appendix A.1.

### 3.4. Variable Measurement

All core constructs were measured using validated scales, adapted to the designer-AI co-creation context. All items employed a 7-point Likert scale (1 = strongly disagree, 7 = strongly agree). Creative control was measured using items adapted from the self-determination dimension of Spreitzer’s (1995) psychological empowerment scale. This dimension specifically assesses individuals’ perceptions of autonomous decision-making in specific work tasks, demonstrating high conceptual alignment with the situational control construct in the present study. The scale comprises three items. A sample item is “In this design process, I was able to control the direction of the design.” Psychological ownership was measured using the scale developed by Pierce et al. (2001), comprising four items. Cognitive load was assessed using F. G. Paas’s (1992) subjective cognitive load scale, comprising three items. Design creativity was measured using items adapted from Tierney and Farmer’s (2002) creative self-efficacy scale, comprising four items. Design experience was measured with a single item assessing years of engagement in design-related work or study (continuous variable). The measurement items for all constructs are summarized in Table 2. The complete list of measurement items is provided in Appendix A.2.

### 3.5. Data Analysis Strategy

Data analysis was conducted using SPSS 26.0 and the PROCESS macro version 3.5 (Hayes, 2013), following a three-stage strategy encompassing measurement model assessment, manipulation check, and structural model testing. In the measurement model assessment stage, scale reliability was evaluated using Cronbach’s α coefficients and composite reliability (CR), with thresholds set at 0.70 (Nunnally, 1978). Convergent validity was assessed through average variance extracted (AVE), with a threshold of 0.50 (Fornell & Larcker, 1981). Discriminant validity was examined through confirmatory factor analysis (CFA), comparing the goodness-of-fit between the four-factor baseline model and nested alternative models. Model fit was evaluated according to the criteria recommended by Hu and Bentler (1999): comparative fit index (CFI) and Tucker–Lewis index (TLI) > 0.90, root mean square error of approximation (RMSEA) and standardized root mean square residual (SRMR) < 0.08. Common method bias was assessed using Harman’s single-factor test (Podsakoff et al., 2003). If the first factor accounted for less than 40% of the variance, serious bias was considered absent. In the manipulation check stage, independent samples t-tests were employed to compare score differences between groups, with *p* < 0.05 and Cohen’s d > 0.50 serving as criteria for successful manipulation (Cohen, 1988). In the structural model testing stage, PROCESS Model 4 was used to test parallel mediation effects, and Model 83 was employed to test moderated mediation effects. Indirect effects were estimated using the bias-corrected bootstrap method (5000 resamples), with 95% confidence intervals not containing zero as the criterion for statistical significance (Preacher & Hayes, 2008). Conditional indirect effects and simple slope analyses were conducted to examine moderation patterns. All analyses controlled for age, gender, and frequency of AI tool usage. The criterion for determining the significance of indirect effects is as follows. The indirect effect (IE) is computed as:

$$\mathrm{IE}\;=\;a\;\times \;b,95\%\;\mathrm{BC}\;\mathrm{CI}\;=\;[\theta_{2.5\%}^{*},\theta_{97.5\%}^{*}]$$

where *a* denotes the regression coefficient from the independent variable (creative control) to the mediator (psychological ownership or cognitive load), and *b* is the regression coefficient from the mediator to the dependent variable (design creativity). Statistical significance is determined by the 95% bias-corrected bootstrap confidence interval [θ*_2.5_, θ*_97.5_], where θ* represents the percentile values obtained from the bootstrap sampling distribution of the indirect effect across 5000 resamples. If this interval excludes zero, the indirect effect is considered statistically significant (Preacher & Hayes, 2008). Moderated mediation was tested using PROCESS Model 83 to analyze the moderating effects of design experience on the two mediation pathways. Simple slope analyses were conducted to illustrate conditional effects at different levels of design experience (±1 SD). Robustness checks were performed through subgroup comparisons between student and professional designer samples to verify the consistency of research findings across different populations.

## 4. Results

This section presents the results of the empirical analyses testing the four hypotheses. The analysis begins with an assessment of measurement quality, including reliability, validity, and common method bias (Section 4.1). This is followed by descriptive statistics and bivariate correlations among the main study variables (Section 4.2). The core hypothesis tests follow, including the dual-pathway mediation analysis (H1 and H2) and the moderated mediation analysis (H3 and H4) in Section 4.3. Finally, robustness checks through subgroup comparisons are presented in Section 4.4. Overall, all four hypotheses received empirical support: creative control exerted a positive indirect effect on design creativity through psychological ownership (H1) and a negative indirect effect through cognitive load (H2), with design experience positively moderating the former pathway (H3) and negatively moderating the latter (H4).

### 4.1. Reliability, Validity, and Common Method Bias Testing

A total of 240 questionnaires were distributed. After excluding 14 invalid responses due to excessively short completion times (<3 min) or obvious patterned responding, 226 valid questionnaires were retained, yielding an effective response rate of 94.2%. The sample comprised 112 participants in the high creative control group and 114 in the low creative control group; the slight between-group difference resulted from the natural distribution following invalid response exclusion. The mean age of participants was 27.3 years (SD = 5.8), with 98 males (43.4%) and 128 females (56.6%). Mean design experience was 4.2 years (SD = 3.6, range: 0.5–18 years). Reliability analysis revealed that Cronbach’s α coefficients for creative control (α = 0.84), psychological ownership (α = 0.89), cognitive load (α = 0.82), and design creativity (α = 0.87) all exceeded 0.80. Composite reliability (CR) values ranged from 0.83 to 0.90, and average variance extracted (AVE) values ranged from 0.62 to 0.69, all meeting recommended thresholds and indicating satisfactory convergent validity. Confirmatory factor analysis results demonstrated that the four-factor model exhibited good fit (χ^2^/df = 1.87, CFI = 0.94, TLI = 0.92, RMSEA = 0.062, SRMR = 0.048) and was significantly superior to the three-factor model (Δχ^2^ = 89.34, Δdf = 3, *p* < 0.001), two-factor model (Δχ^2^ = 186.52, Δdf = 5, *p* < 0.001), and single-factor model (Δχ^2^ = 312.67, Δdf = 6, *p* < 0.001), indicating satisfactory discriminant validity among all variables. Harman’s single-factor test revealed that the first unrotated factor accounted for 32.4% of the variance, below the 40% threshold, suggesting that common method bias was not a serious concern. Independent samples t-test results indicated that the high creative control group scored significantly higher on creative control (M = 5.42, SD = 0.98) than the low creative control group (M = 3.71, SD = 1.12), t(224) = 12.36, *p* < 0.001, Cohen’s d = 1.63. The effect size reached a large effect level, confirming successful experimental manipulation. Detailed reliability and validity results are presented in Table 3.

### 4.2. Descriptive Statistics and Correlation Analysis

Table 4 presents the means, standard deviations, and correlation coefficients of the main study variables. Results revealed that creative control was significantly positively correlated with psychological ownership (r = 0.41, *p* < 0.001) and cognitive load (r = 0.28, *p* < 0.001), providing preliminary support for the theoretical hypothesis that creative control simultaneously activates both pathways. Psychological ownership was significantly positively correlated with design creativity (r = 0.43, *p* < 0.001), while cognitive load was significantly negatively correlated with design creativity (r = −0.32, *p* < 0.001), consistent with theoretical expectations. Design experience was significantly positively correlated with psychological ownership (r = 0.19, *p* < 0.01) and design creativity (r = 0.24, *p* < 0.001), and significantly negatively correlated with cognitive load (r = −0.16, *p* < 0.05), providing preliminary evidence for subsequent moderation analyses. All inter-variable correlation coefficients were below 0.50, indicating the absence of serious multicollinearity concerns.

### 4.3. Hypothesis Testing

**Figure 2:**
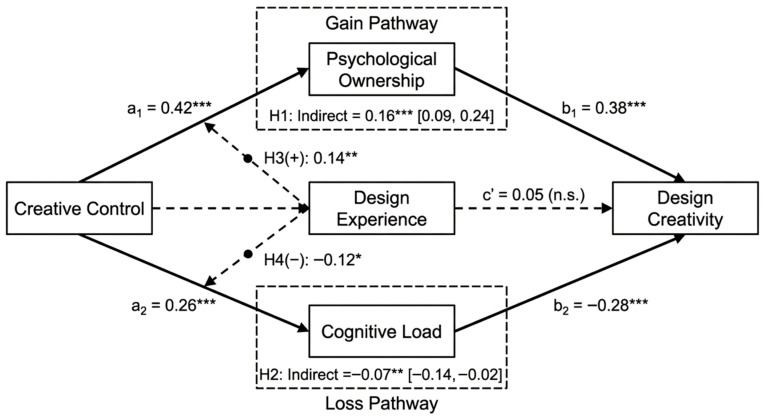
Path Coefficient Results. Note: *** *p* < 0.001, ** *p* < 0.01, * *p* < 0.05, n.s. = not significant. Values in brackets are 95% bootstrap confidence intervals.

Parallel mediation effects of psychological ownership and cognitive load were tested using PROCESS Model 4, with 5000 bootstrap resamples and 95% confidence intervals, controlling for age, gender, and frequency of AI tool usage. Results indicated that the total effect of creative control on design creativity was significant (c = 0.14, SE = 0.06, 95% CI = [0.02, 0.26]). However, after including both mediators, the direct effect became non-significant (c′ = 0.05, SE = 0.05, 95% CI = [−0.07, 0.13]), suggesting full mediation. Regarding the positive pathway, creative control exerted a significant positive effect on psychological ownership (a_1_ = 0.42, SE = 0.06, *p* < 0.001), and psychological ownership exerted a significant positive effect on design creativity (b_1_ = 0.38, SE = 0.05, *p* < 0.001). The indirect effect through psychological ownership was significant (a_1_b_1_ = 0.16, Boot SE = 0.04, 95% CI = [0.09, 0.24]), supporting Hypothesis 1. Regarding the negative pathway, creative control exerted a significant positive effect on cognitive load (a_2_ = 0.26, SE = 0.07, *p* < 0.001), and cognitive load exerted a significant negative effect on design creativity (b_2_ = −0.28, SE = 0.05, *p* < 0.001). The indirect effect through cognitive load was significant (a_2_b_2_ = −0.07, Boot SE = 0.03, 95% CI = [−0.14, −0.02]), supporting Hypothesis 2. The contrast test of the two indirect effects revealed that the positive pathway indirect effect was significantly larger than the negative pathway (difference = 0.23, Boot SE = 0.05, 95% CI = [0.14, 0.33]), indicating that the positive effect of creative control through psychological ownership predominated in the overall sample. Detailed mediation analysis results are presented in Table 5, and path coefficient results are illustrated in Figure 2.

PROCESS Model 83 was employed to further examine the moderating effects of design experience on the two mediation pathways. Results indicated that the interaction term between creative control and design experience exerted a significant positive effect on psychological ownership (β = 0.14, SE = 0.05, t = 2.78, *p* = 0.006, 95% CI = [0.04, 0.24]), suggesting that design experience positively moderated the relationship between creative control and psychological ownership. Hypothesis 3 was supported. The interaction term between creative control and design experience exerted a significant negative effect on cognitive load (β = −0.12, SE = 0.06, t = −2.14, *p* = 0.033, 95% CI = [−0.23, −0.01]), suggesting that design experience negatively moderated the relationship between creative control and cognitive load. Hypothesis 4 was supported. Detailed results of the moderated mediation analysis are presented in Table 6.

**Figure 3:**
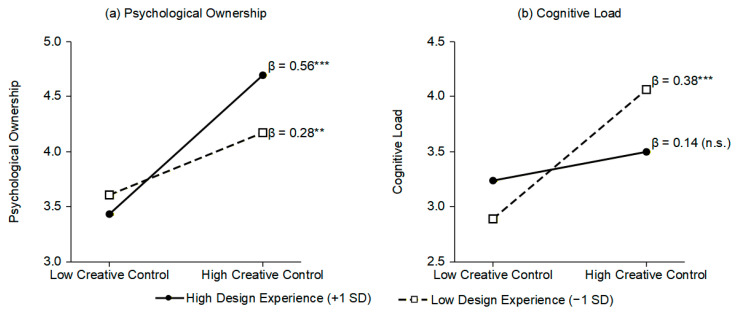
Moderating Effects of Design Experience on the Relationships between Creative Control and Psychological Ownership/Cognitive Load. Note: (**a**) The relationship between creative control and psychological ownership; (**b**) The relationship between creative control and cognitive load. Solid lines represent high design experience (+1 SD), dashed lines represent low design experience (−1 SD). β indicates simple slope coefficients. ** *p* < 0.01, *** *p* < 0.001, n.s. = not significant.

Simple slope analysis further revealed the specific patterns of moderating effects. For the positive pathway (creative control → psychological ownership), the slope for high design experience (+1 SD) was 0.56 (*p* < 0.001), for mean level was 0.42 (*p* < 0.001), and for low design experience (−1 SD) was 0.28 (*p* = 0.004). These results indicate that greater design experience strengthens the positive effect of creative control on psychological ownership. For the negative pathway (creative control → cognitive load), the slope for high design experience was 0.14 (*p* = 0.089, non-significant), for mean level was 0.26 (*p* = 0.002), and for low design experience was 0.38 (*p* < 0.001). These results indicate that greater design experience weakens the positive effect of creative control on cognitive load. The visualization of moderating effects is presented in Figure 3. Conditional indirect effect analysis revealed that at high levels of design experience, the indirect effect through psychological ownership was stronger (effect = 0.24, 95% CI = [0.15, 0.35]), whereas the indirect effect through cognitive load was non-significant (effect = −0.04, 95% CI = [−0.11, 0.02]). At low levels of design experience, the indirect effect through psychological ownership was attenuated (effect = 0.12, 95% CI = [0.03, 0.22]), whereas the indirect effect through cognitive load was enhanced (effect = −0.11, 95% CI = [−0.20, −0.04]). These findings suggest that highly experienced designers can more effectively convert creative control into psychological ownership while simultaneously buffering the negative effects of cognitive load. In contrast, less experienced designers, although able to obtain certain psychological ownership benefits, bear greater cognitive load costs.

### 4.4. Robustness Checks

To verify the robustness of research findings, the sample was divided into student (*n* = 108) and professional designer (n = 118) subsamples for separate analyses. H1, H2, and H4 were consistently supported across both subsamples. H3 was supported in the professional designer subsample (β = 0.16, *p* = 0.012), while in the student subsample, the effect direction was consistent but did not reach conventional significance levels (β = 0.11, *p* = 0.067). This may be attributable to the relatively narrower range of design experience variance in the student sample (student sample SD = 2.1 years vs. professional designer sample SD = 4.2 years), which limited the statistical power to detect the moderation effect. Overall, the core dual-pathway mediation effects (H1, H2) demonstrated satisfactory cross-sample robustness, whereas the moderating effect of design experience on the psychological ownership pathway (H3) requires further validation in samples with broader experience distributions. In summary, all four hypotheses were supported by empirical evidence: psychological ownership positively mediates the relationship between creative control and design creativity (H1); cognitive load negatively mediates this relationship (H2); design experience positively moderates the relationship between creative control and psychological ownership (H3); and design experience negatively moderates the relationship between creative control and cognitive load (H4).

## 5. Discussion

Drawing on a scenario experiment with 226 designers and design students, the dual-pathway mechanism by which creative control influences design creativity in designer-AI co-creation was validated. The findings are discussed below in relation to the two research questions guiding this study.

### 5.1. The Dual-Pathway Effect of Creative Control (RQ1)

The first research question investigated whether creative control exerts simultaneous positive and negative indirect effects on design creativity through psychological ownership and cognitive load, respectively. The results confirm this dual-pathway mechanism. Creative control simultaneously activates psychological ownership (a_1_ = 0.42) and cognitive load (a_2_ = 0.26), shaping creativity through competing “gain” and “loss” pathways. This finding aligns with Yin et al. (2024) research on the double-edged sword effect of AI assistants, extending that logic to control allocation in human-AI collaboration. From a Conservation of Resources perspective (Hobfoll, 1989), creative control, as a psychological resource, shows context-dependent utility, stimulating ownership while potentially triggering excessive cognitive consumption.

The positive pathway’s larger indirect effect (0.16 vs. −0.07) merits deeper examination. One explanation concerns the scenario-based design, in which participants imagined rather than experienced cognitive demands, a methodological feature that may have attenuated the cognitive load pathway relative to real-world settings. Alternatively, psychological ownership, as an affective-motivational resource, may exert a stronger influence on creative self-assessment than the inhibitory effects of cognitive load. This interpretation aligns with Wadinambiarachchi et al. (2024), who found stronger negative effects when studying actual designer-AI dependence rather than perceived control, suggesting that real interaction may amplify the cognitive load pathway beyond what scenario methods capture. Zhu et al. (2024) observed similar dual-pathway mechanisms for control perception in platform work contexts, providing cross-context support for the competing mediation framework. Multiple studies support psychological ownership as a positive mediator: Gray et al. (2020) revealed how collective ownership promotes creative success; Baer and Brown (2012) found that ownership enhances willingness to adopt suggestions; Pierce et al. (2003) clarified control’s role in ownership formation. This study extends the ownership context from traditional organizations to human-AI collaboration, aligning with Ryan and Deci’s (2000) proposition that autonomy promotes intrinsic motivation. For cognitive load’s negative mediation, Rodet (2022) documented nonlinear load-creativity relationships, and F. Paas et al. (2016) emphasized the complex associations between load levels and task performance.

### 5.2. The Moderating Role of Design Experience (RQ2)

The second research question examined whether design experience moderates the dual pathways. The results reveal a clear differential pattern: experienced designers effectively convert control into ownership (slope = 0.56) while buffering cognitive load effects (slope = 0.14, non-significant). Less experienced designers obtain ownership benefits (slope = 0.28) but bear substantially greater cognitive costs (slope = 0.38). This asymmetry reflects underlying differences in cognitive architecture. Experienced designers possess automated procedural knowledge—what Dreyfus and Dreyfus (1986) term “intuitive expertise”—enabling pattern recognition-based decisions that consume minimal working memory. When such designers maintain high creative control, many evaluative and directional decisions proceed quasi-automatically, leaving cognitive capacity available for creative exploration. Novices, by contrast, rely on resource-intensive analytical processing for each decision, making them more vulnerable to overload as control demands increase. This interpretation is consistent with Chen et al.’s (2022) demonstration that expert designers encode and reorganize design information through more efficient knowledge structures, and with the expertise reversal effect documented by Kalyuga (2007), which predicts that instructional conditions beneficial for novices may become redundant or counterproductive for experts. Ozkan and Dogan’s (2013) research on cognitive strategy differences in analogical design reasoning further corroborates this pattern: experts rapidly identify structural connections between solutions and intentions, while novices invest greater effort in surface-level analysis.

### 5.3. Theoretical and Practical Implications

Theoretically, this study extends Conservation of Resources theory from organizational contexts to human-AI collaboration by constructing an integrated “creative control → psychological ownership/cognitive load → design creativity” model. Unlike Verganti et al.’s (2020) optimistic perspective or concerns about AI diminishing creativity, this study integrates these contradictory viewpoints through competing mediation, thereby characterizing control’s “gains” and “losses.” Pierce et al. (2001) emphasized control as a core ownership pathway; these findings validate this pathway’s effectiveness in AI collaboration contexts, responding to Zhang et al.’s (2021) call for attention to contextual factors in ownership research. Lubart (2005) explored computers as creativity partners; this study provides new evidence from a control allocation perspective. Practically, these findings offer guidance for AI-assisted design tool development: differentiated strategies can optimize human-AI collaboration experience, consistent with Shneiderman’s (2022) philosophy of “human-centered AI”.

### 5.4. Limitations and Future Directions

Several limitations warrant acknowledgment. Methodologically, the scenario design required participants to imagine AI collaboration by reading ~200-word descriptions rather than actually interacting with tools like Midjourney or DALL-E. Consequently, the study measured “imagined” rather than “experienced” creative control—a distinction that merits attention in future research. Future studies could employ field experiments or longitudinal studies with actual AI tools to enhance ecological validity. For measurement, design creativity was assessed using self-report scales susceptible to social desirability bias, and experience was operationalized using years as a single indicator, potentially omitting multidimensional information such as project quantity or portfolio quality. Regarding sampling, voluntary uncompensated participation may have attracted individuals with positive AI attitudes, creating self-selection bias. The sample’s positively skewed experience distribution (M = 4.2, SD = 3.6 years), with few senior designers (10+ years), limited power to detect high-experience effects and generalizability to senior populations. Theoretically, the study focused on two mediation pathways; future research could incorporate creative self-efficacy, flow experience, and other constructs. Contextually, the study examined product aesthetic design; generalization to other design domains and cross-cultural contexts requires careful validation.

## 6. Conclusions

Grounded in Conservation of Resources theory, this study built a competing mediation model examining how creative control influences design creativity in designer-AI co-creation. Using scenario-based experimental data from 226 designers and design students, the study validated creative control’s “double-edged sword” mechanism: a positive indirect effect through psychological ownership (effect = 0.16) and a negative indirect effect via cognitive load (effect = −0.07), with design experience differentially moderating both pathways. These findings contribute to several ongoing scholarly conversations. Within human–computer interaction (HCI), the dual-pathway model moves beyond usability-centered evaluations toward understanding the psychological mechanisms underlying human-AI creative collaboration (Koch et al., 2019; Caramiaux et al., 2025). Within design cognition, the moderation findings extend the expert-novice paradigm (Cross, 2023; Chen et al., 2022) to human-AI collaborative contexts, providing empirical evidence for what expertise concretely affords in AI-mediated creative work. Within human-AI collaboration research, this study complements Verganti et al. (2020) and Shneiderman (2022) by demonstrating that effective collaboration requires differentiated rather than uniform control allocation. Practically, system designers should implement experience-adaptive interfaces: higher creative control for experienced designers, and more structured cognitive scaffolding with progressive autonomy for less experienced users.

## Figures and Tables

**Table 1:** Comparison of Experimental Materials.

Design Element	High Creative Control Group	Low Creative Control Group	Theoretical Basis
Design Direction	Designer autonomously sets style parameters, material constraints, and morphological features	AI autonomously analyzes market trends and user preferences	Ryan and Deci (2000); Pierce et al. (2001)
Solution Generation	AI generates alternative solutions based on designer’s specific instructions	AI automatically generates diverse design solutions	Woodbury and Burrow (2006); Koch et al. (2019)
Iterative Decision-Making	Designer holds decision-making authority over iteration direction	Designer selects and fine-tunes from AI-generated solutions	Sweller (1988); F. Paas et al. (2016)
Role Positioning	Designer-led, AI assists in execution	AI-led exploration, designer screens and confirms	Lubart (2005); Verganti et al. (2020)
Scenario Description Length	Approximately 200 words	Approximately 200 words (identical)	—
Design Task	Smart speaker aesthetic design	Smart speaker aesthetic design (identical)	—
AI Tool Type	Generative AI design tool	Generative AI design tool (identical)	—

*Note*: The two scenario conditions were completely identical in terms of task content, product type, and information quantity; the sole difference lay in the allocation of control.

**Table 2:** Variable Measurement Scales.

Construct	Number of Items	Sample Item	Source
Manipulation Check: Creative Control	3	“In this design process, I was able to control the direction of the design”	Adapted from Spreitzer (1995)
Psychological Ownership	4	“I feel that this design work is ‘mine’“	Pierce et al. (2001)
Cognitive Load	3	“In this design process, I felt a heavy mental burden”	F. G. Paas (1992)
Design Creativity	4	“I believe I demonstrated creativity in this task”	Adapted from Tierney and Farmer (2002)
Design Experience	1	Years of engagement in design-related work or study (continuous variable)	Self-developed

*Note*: Control variables include age, gender, and frequency of AI tool usage.

**Table 3:** Reliability and Validity Test Results.

Variable	Number of Items	Cronbach’s α	CR	AVE
Creative Control	3	0.84	0.85	0.66
Psychological Ownership	4	0.89	0.90	0.69
Cognitive Load	3	0.82	0.83	0.62
Design Creativity	4	0.87	0.88	0.65

*Note*: CR = composite reliability; AVE = average variance extracted.

**Table 4:** Descriptive Statistics and Correlation Matrix (N = 226).

Variable	M	SD	1	2	3	4	5
1. Design Experience	4.20	3.60	—				
2. Creative Control	4.56	1.32	0.12	—			
3. Psychological Ownership	4.38	1.18	0.19 **	0.41 ***	—		
4. Cognitive Load	3.72	1.24	−0.16 *	0.28 ***	−0.08	—	
5. Design Creativity	4.61	1.08	0.24 ***	0.18 **	0.43 ***	−0.32 ***	—

*Note*: * *p* < 0.05, ** *p* < 0.01, *** *p* < 0.001.

**Table 5:** Mediation Effect Test Results (Bootstrap N = 5000).

Effect Path	Effect	Boot SE	95% CI	Conclusion
Total effect (c)	0.14	0.06	[0.02, 0.26]	Significant
Direct effect (c′)	0.05	0.05	[−0.07, 0.13]	Non-significant
Indirect effect 1: Creative Control → Psychological Ownership → Design Creativity	0.16	0.04	[0.09, 0.24]	H1 Supported
Indirect effect 2: Creative Control → Cognitive Load → Design Creativity	−0.07	0.03	[−0.14, −0.02]	H2 Supported
Effect contrast (Indirect effect 1 − Indirect effect 2)	0.23	0.05	[0.14, 0.33]	Significant difference

*Note*: Control variables include age, gender, and frequency of AI tool usage. Significance of indirect effects was determined based on whether the 95% bias-corrected bootstrap confidence interval contained zero.

**Table 6:** Moderated Mediation Effect Test Results.

Interaction Term	β	SE	t	*p*	95% CI	Conclusion
Creative Control × Design Experience → Psychological Ownership	0.14 **	0.05	2.78	0.006	[0.04, 0.24]	H3 Supported
Creative Control × Design Experience → Cognitive Load	−0.12 *	0.06	−2.14	0.033	[−0.23, −0.01]	H4 Supported

*Note*: Control variables include age, gender, and frequency of AI tool usage; * *p* < 0.05, ** *p* < 0.01.

## Data Availability

The data presented in this study are available on request from the corresponding author due to privacy restrictions.

## Appendix A. Experimental Materials

### Appendix A.1. Scenario Descriptions

Participants were randomly assigned to one of the following two scenario conditions. Both scenarios described a designer using a generative AI tool to explore the aesthetic design of a smart speaker product. The scenarios were matched in word count, task content, and product type, differing only in the allocation of creative control.

#### Appendix A.1.1. High Creative Control Condition

Imagine you are a product designer working on the aesthetic design of a new smart speaker. You are using a generative AI design tool to explore the design space. In this process, you take the lead in setting the overall design direction. You autonomously define the style parameters, including the form language, material constraints, and key morphological features of the product. Based on your specific creative instructions, the AI tool generates a series of design alternatives that align with your defined direction. As the design progresses, you evaluate each AI-generated solution against your creative vision and make all iterative decisions—determining which elements to retain, modify, or discard. You decide when the design has reached a satisfactory level of refinement. Throughout the process, you are the primary driver of the creative direction, and the AI serves as an assistive tool that executes and visualizes your design intentions.

#### Appendix A.1.2. Low Creative Control Condition

Imagine you are a product designer working on the aesthetic design of a new smart speaker. You are using a generative AI design tool to explore the design space. In this process, the AI tool takes the lead in setting the exploration direction. The AI autonomously analyzes current market trends, user preferences, and design best practices, and then automatically generates a diverse set of design solutions covering different styles, materials, and morphological features. Your role is to browse through the AI-generated alternatives, select the solutions that you find most promising, and provide feedback for fine-tuning. The AI incorporates your selections and feedback to refine and generate further iterations. The AI determines the scope and direction of exploration, while you are responsible for screening, evaluating, and confirming the final output. Throughout the process, the AI is the primary driver of the creative exploration, and you serve as an evaluator and selector who guides the process through your choices.

### Appendix A.2. Measurement Items

All items were measured on a 7-point Likert scale (1 = strongly disagree, 7 = strongly agree).

#### Appendix A.2.1. Manipulation Check: Creative Control (Adapted from Spreitzer, 1995)

1. In this design process, I was able to control the direction of the design.
2. In this design process, I had considerable autonomy in determining how the design work was carried out.
3. In this design process, I could decide on my own how to approach the design task.

#### Appendix A.2.2. Psychological Ownership (Adapted from Pierce et al., 2001)

1. I feel that this design work is “mine.”
2. I feel a strong sense of personal connection to the design outcomes.
3. I feel that I have a significant personal stake in the design results.
4. I feel that the design outcomes reflect my personal creative input.

#### Appendix A.2.3. Cognitive Load (Adapted from F. G. Paas, 1992)

1. In this design process, I felt a heavy mental burden.
2. The design task required a great deal of mental effort.
3. I found the design process to be mentally demanding.

#### Appendix A.2.4. Design Creativity (Adapted from Tierney & Farmer, 2002)

1. I believe I demonstrated creativity in this task.
2. I feel that I came up with novel ideas during this design process.
3. I think the design outcomes reflect creative thinking.
4. I feel that I approached the task in an innovative way.

#### Appendix A.2.5. Design Experience

1. How many years have you been engaged in design-related work or study? ______ years (continuous variable)

#### Appendix A.2.6. Control Variables

1. Age: ______ years
2. Gender: Male/Female/Other
3. How frequently do you use AI-assisted design tools? (1 = never, 7 = very frequently)
